# Geriatric nutritional risk index predicts all-cause mortality in the oldest-old patients with acute coronary syndrome: A 10-year cohort study

**DOI:** 10.3389/fnut.2023.1129978

**Published:** 2023-03-07

**Authors:** Ying Li, Jian Shen, Xiaoling Hou, Yongkang Su, Yang Jiao, Jihang Wang, Henan Liu, Zhenhong Fu

**Affiliations:** ^1^Senior Department of Cardiology, The Sixth Medical Center, Chinese PLA General Hospital, Beijing, China; ^2^Chinese PLA Medical School, Beijing, China; ^3^Department of Cardiology, Hainan Hospital, Chinese PLA General Hospital, Sanya, China

**Keywords:** oldest old, acute coronary syndrome, malnutrition, GNRI, mortality

## Abstract

**Background and objective:**

Nutritional status assessment in acute coronary syndrome (ACS) patients has been neglected for a long time. The geriatric nutritional risk index (GNRI) is a sensitive indicator for assessing the nutritional status of the elderly. This study aims to explore the association between GNRI and all-cause mortality in the oldest-old patients with ACS.

**Methods:**

The patients who met the inclusion criteria were consecutively enrolled from January 2006 to December 2012. Clinical data were collected on admission, and all subjects were followed after being discharged. The nutritional status was evaluated using GNRI. The relationship between GNRI and all-cause mortality was assessed by using different analyses.

**Results:**

A total of 662 patients with a mean age of 81.87 ± 2.14 years old were included in our study, and followed (median: 63 months, IQR 51–71). Patients whose GNRI ≤ 98 were reported as at risk of malnutrition (31.11%, *n* = 206). In multivariable analysis, we found that for each SD increase in GNRI, the risk of all-cause mortality lowered by 23%, and the HR for GNRI ≤ 98 was 1.39 (95% CI 1.04–1.86). After stratifying patients into three groups by tertiles of GNRI, we found that the HRs for tertile 2 and tertile 3 were 1.49 (95% CI 1.02–2.19) and 1.74 (95% CI 1.22–2.50), respectively. The trend test revealed a dose–response relationship between GNRI and all-cause mortality in the oldest-old with ACS. Lastly, in subgroup analyses, we found a reliable association between GNRI and all-cause mortality.

**Conclusion:**

Malnutrition is common in the oldest-old patients with ACS, and GNRI could predict their long-term all-cause mortality in a dose-dependent manner. GNRI may be a prospective index for risk-stratification and secondary-prevention in the oldest-old patients with ACS.

## 1. Introduction

Acute coronary syndrome (ACS) is one of the leading causes of morbidity and mortality worldwide (1, 2). Aging is a vital risk factor for its prevalence and poor clinical outcomes. Almost a third of patients admitted for ACS and two-thirds of those dying from ACS are >75 years old (3, 4). Multi-comorbidities, complicated coronary artery lesions, and high prevalence of frailty in the oldest-old have increased the risk of re-infarcted, bleeding complications, and mortality when compared to younger patients (5–7).

Malnutrition is a common but under-recognized problem in hospitalized patients and caused detrimental and extensive impacts on clinical results with negative and far-reaching consequences for clinical outcomes (8, 9). As estimated, about 30–60% of hospitalized patients are malnourished. It not only leads to a high economic burden but is associated with longer hospital stays and higher mortality (10, 11). A higher prevalence of malnutrition has been found in the elderly (12). Recently, the side effects of malnutrition in cardiovascular diseases have come under the researchers’ spotlight. Clinical studies have demonstrated that malnutritional status has negative effects on people affected by cardiovascular diseases including ACS (13–16).

Geriatric nutritional risk index (GNRI) was first created by Bouillanne et al. to identify nutritional-related complications among the elderly (17–19). It has been used to assess the nutritional status of patients with heart failure (20), chronic kidney disease (CKD) (21), tumors (22), etc. And many studies have revealed that GNRI was significantly associated with vascular calcification, length of hospital stay, and mortality (23, 24). However, studies about the relationship between GNRI and the prognosis of ACS have seldom been conducted (25).

Herein our study aimed to explore the relationship between GNRI and all-cause mortality of the oldest-old ACS patients, investigate the predictive value of GNRI on patients; long-term prognosis, and assess the effectiveness of the risk-stratify for them.

## 2. Methods

### 2.1. Study design and population

**Figure 1:**
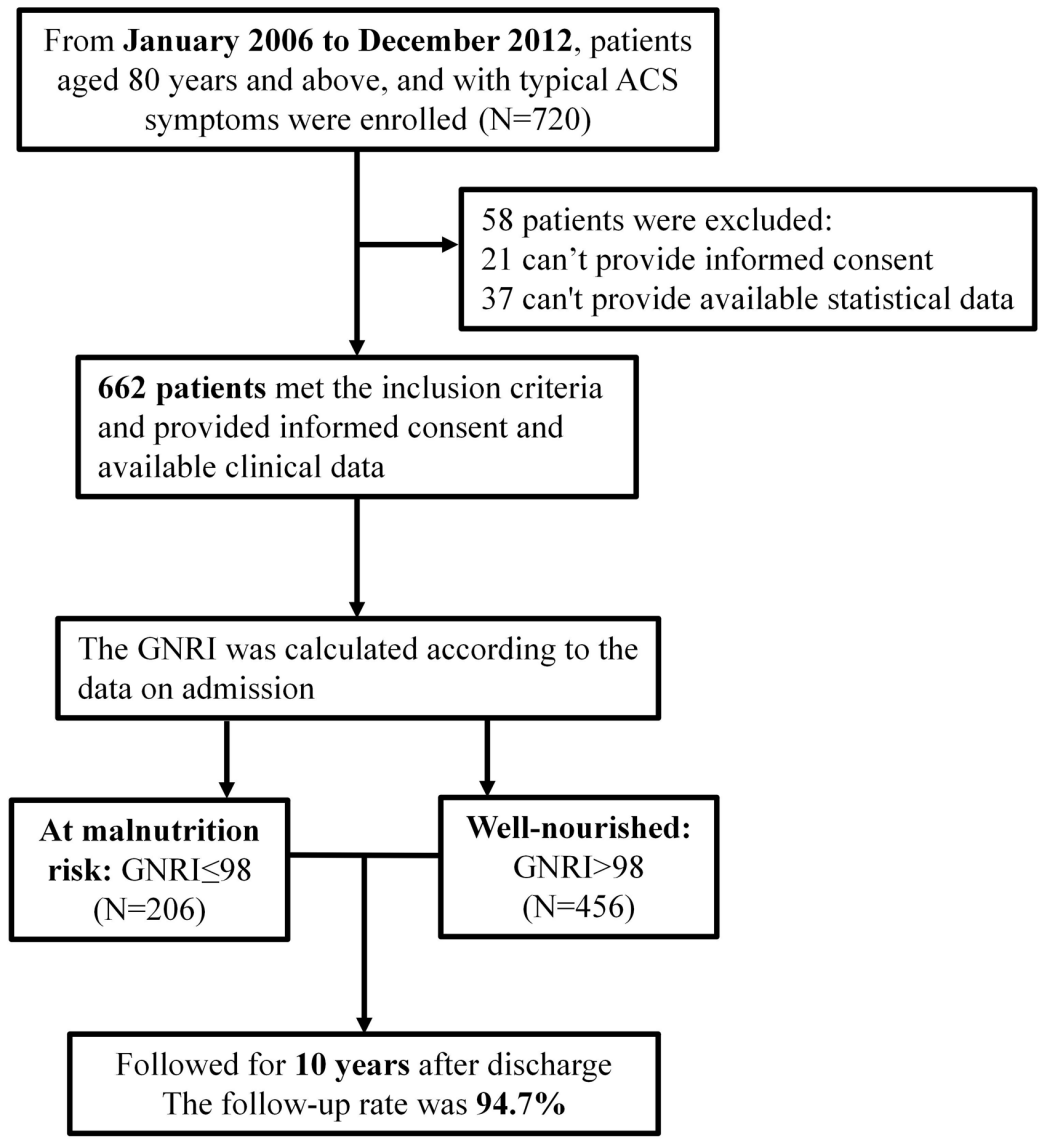
Flow chart of patient enrollment, grouping, and follow-up.

From January 2006 to December 2012, 720 patients aged ≥80 admitted to the cardiology department of the Chinese People’s Liberation Army (PLA) General Hospital for coronary angiography due to ACS symptoms, were enrolled (Figure 1). A total of 699 patients signed their informed consent. The exclusion criteria were: (1) patients with severe valvular heart disease, severe pulmonary hypertension, severe liver or renal insufficiency, rheumatoid arthritis, infectious diseases, and malignant tumors; (2) patients with familial hypertriglyceridemia (triglyceride ≥5.65 mmol/L); and (3) patients with neuropsychiatric problems that prevent them cooperating with the researchers. Most critically, our study followed the Declaration of Helsinki and was certified by the Chinese PLA General Hospital’s Ethics Service Center.

To confirm the diagnosis of coronary heart disease, the cardiac intervention center of the PLA General Hospital performed coronary intervention and perioperative treatment according to current guidelines. All of the angiography results were analyzed using the same image analysis tool. Loading doses of aspirin (300 mg) and clopidogrel (300 mg) were administered before the intervention. The degree of coronary stenosis was determined using the Gensini score (26), and two experienced experts trained the recorders. Individualized interventions, such as intensive medication therapy, percutaneous coronary intervention (PCI), or coronary artery bypass grafting (CABG), were administered based on coronary angiography results, and long-term follow-up was conducted after being discharged.

### 2.2. Data collection and index evaluation

We collected demographic data (age and gender), anthropometric data [height, body weight, BMI, heart rate, systolic blood pressure (SBP), and diastolic blood pressure (DBP)], laboratory data [total cholesterol (TC), triglycerides (TG), low-density lipid-cholesterol (LDL-C), high-density lipid-cholesterol (HDL-C), estimated glomerular filtration rate (eGFR), fasting plasma glucose (FPG), uric acid (UA), albumin], left ventricular ejection fraction (LVEF), comorbidity data [diabetes mellitus type 2 (T2DM), hypertension, stroke, prior myocardial infarction (MI), hyperlipidemia and chronic kidney disease (CKD)], smoking, drug-usage data [aspirin, clopidogrel, statins, β-blocker and angiotensin-converting enzyme inhibitor (ACEI)/angiotensin receptor blocker (ARB)], coronary lesions data[left anterior descending branch (LAD), left circumflex branch (LCX), right coronary artery (RCA), left main coronary artery (LM), multivessel lesions and Gensini score] and treatment data (intensive medications, PCI and CABG).

The body mass index (BMI) was calculated as follows:


$$\mathrm{BMI}=\mathrm{height}\;\left(m^{2}\right)/{\left[\mathrm{weight}\;\left(\mathrm{kg}\right)\right]}^{2}$$

According to WHO criteria in the Asian population, patients could be classified as overweight (BMI > 24.9 kg/m^2^), normal-weight (BMI 18.5–24.9 kg/m^2^), and under-weight (BMI < 18.5 kg/m^2^) (27).

The eGFR was calculated by the Chinese modified Modification of Diet in Renal Disease equation:


$$\begin{array}{l} \mathrm{eGFR}\;\left(\mathrm{ml}/\min/1.73m^{2}\right)=\\ \;\;\;\;\;\;\;\;\;\;\;\;\;\;\;\;\;\;\;\;\;\;\;\;\;\;\;\;\;\;\;\;\;\;\;175\times \mathrm{standardlized creatinine}\;{\left(\mathrm{mg}/\mathrm{dl}\right)}^{-1.234}\times \\ \;\;\;\;\;\;\;\;\;\;\;\;\;\;\;\;\;\;\;\;\;\;\;\;\;\;\;\;\;\;\;\;\;\;\;\mathrm{age}\;{\left(\mathrm{years}\;\mathrm{old}\right)}^{-0.179}\times 0.79 \end{array}$$

Standardized creatinine (Scr) was calculated by the calibration equation:


Scr(mg/dl)=0.795×[enzymatic methodScr(mg/dl)]+0.29

Chronic kidney disease was defined as eGFR <60 ml/min/1.73 m^2^.

The diagnostic criteria for diabetes mellitus type 2 (T2DM) were: FPG ≥ 7.0 mmol/L; and (or) random blood glucose (RBG) ≥ 11.1 mmol/L; blood glucose ≥11.1 mmol/L 2 h after oral glucose tolerance test (OGTT).

Hyperlipidemia was defined as the use of lipid-lowering drugs or total serum cholesterol ≥240 mg/dl.

Based on coronary angiography results, the multivessel lesion was defined as having more than 2 vessels with significant diameter stenosis of 50%.

### 2.3. Assessment of nutritional status

The geriatric nutritional risk index (GNRI) was used in this study to assess the nutritional status of the oldest-old patients with ACS. GNRI is calculated as follow (17):


$$\begin{array}{l} \mathrm{GNRI}=1.489\times \mathrm{albumin}\;\left(g/L\right)+41.7\times \\ \;\;\;\;\;\;\;\;\;\;\;\;\;\;\;\;\;\;\;\left(\mathrm{weight}/\mathrm{ideal weight}\right)\;\left(\mathrm{kg}\right) \end{array}$$

The ideal weight was calculated as follows: 22 × square of height (m^2^) (18). It is worth noting that GNRI was created to identify and predict nutritional-related complications (17). The original GNRI cut-off values and grades of nutrition-related risk were: severe risk (GNRI <82), moderate risk (GNRI 82–92), low risk (GNRI 92–98), and no risk (GNRI >98). Patients were often considered as having a normal nutritional status if their GNRI >98 (19). Hence, we used 98 as the cut-off value in the present study. GNRI >98 was defined as well-nourished, while GNRI≤98 was defined as at malnutrition risk.

### 2.4. Endpoint and follow-up

The follow-up period lasted up to 10 years performed every 12 months after discharge *via* outpatient visits, telephone records, or medical records of outcomes. During the follow-up period, 37 patients were lost follow-up, leaving 662 (94.7%) patients enrolled in the final statistical analysis. All-cause mortality (cardiac and non-cardiac) was the ultimate endpoint of our research.

### 2.5. Statistical analysis

The baseline characteristics of the participants were shown according to GNRI >98 and GNRI ≤98. The measurement data of normal distribution were expressed as mean ± SD, and the *T*-test was used for homogeneity of variance. If the variance is not uniform, the rank-sum test was used. Non-normally distributed measurements were represented by the median and interquartile range (IQR). Statistical data were expressed by quantity, the chi-square test was used to evaluate differences between groups, and an analysis of variance was used to compare data between groups. Pearson correlation test was used to evaluate the correlation between GNRI and clinical parameters. Unadjusted survival curves were generated using log-rank tests in Kaplan–Meier plots. Univariate Cox regression analysis (HR, 95% CI) was used to identify the factors associated with all-cause mortality. *p* < 0.05 was considered statistically significant. The Cox proportional hazards model was used to estimate the association between GNRI and all-cause mortality. We built three regression models: model 1 is the unadjusted model, model 2 is the partially adjusted model (age and gender), and model 3 is the completely adjusted model (age, gender, diabetes, stroke, CKD, aspirin, eGFR, FPG, UA, EF, Gensini score, LM lesions, multivessel lesions, and HDL-C). The GNRI was transformed into three classification variables for the primary analysis. For the trend test, the new categorical variables were recorded as continuous variables and entered into the regression model. We also standardized the GNRI and then put it into a regression model to determine the relationship between the change in GNRI per SD and all-cause mortality. In addition, we performed a subgroup analysis to explore whether the relationship between GNRI and all-cause mortality could be modified by the following variables: gender, diabetes, hypertension, prior MI, hyperlipidemia, CKD, smoking, LM lesions, and multivessel lesions. Interactions between GNRI and the above variables were tested. Results were reported as HR and 95% CI. Two-sided *p* < 0.05 was considered statistically significant. All analyses were performed using the statistical software packages R (28) and Empower Stats (29).

## 3. Results

### 3.1. Baseline characteristics and malnutrition assessment

There were 662 oldest-old patients with ACS enrolled in our study, 71.9% (*n* = 476) of whom were males (Table 1). The mean age of the participants was 81.87 ± 2.14 years old (IQR 80–89), and the average GNRI at admission was 102.47 ± 11.06. According to GNRI, the patients were classified as well-nourished (GNRI >98, *n* = 456, 68.89%), and at malnutrition risk (GNRI ≤98, *n* = 206, 31.11%). Patients with low GNRI had lower body weight, BMI, SBP, LVEF, albumin, and eGFR, while they have higher height and heart rate. There were no statistical differences in TC, TG, LDL-C, HDL-C, FPG, and UA between the two groups. Patients at malnutrition risk had a higher proportion of CKD but a lower proportion of hypertension. There was no significant difference in the prevalence of other common complications such as diabetes, hyperlipidemia, or stroke. What is more, we noticed that patients with low GNRI were more likely to have RCA lesions and multivessel lesions, and got a higher Gensini score. In terms of smoking behavior, medication usage, and treatment manners, there was no significant difference between the two groups.

**Table 1:** Baseline characteristics of all participants (*N* = 662).

Variable	Total	GNRI >98	GNRI ≤98	*d*-value	*p-*Value
*N*	662	456	206		–
Demographic data					
Age (year old)	81.87 ± 2.14	81.83 ± 2.11	81.98 ± 2.20	−0.0701	0.398
Female	186 (28.10%)	135 (29.61%)	51 (24.76%)	0.1079	0.199
Anthropometric data					
Height (cm)	165.32 ± 8.25	164.81 ± 8.61	166.46 ± 7.27	−0.1806	0.017
Body weight (kg)	67.20 ± 10.68	69.74 ± 10.16	61.55 ± 9.63	0.8191	<0.001
BMI (kg/m^2^)	24.57 ± 3.40	25.64 ± 2.97	22.19 ± 3.08	1.1482	<0.001
Heart rate (bpm)	74.81 ± 14.02	74.01 ± 13.47	76.57 ± 15.07	−0.183	0.029
SBP (mmHg)	137.08 ± 21.79	138.41 ± 21.10	134.15 ± 23.02	0.1962	0.020
DBP (mmHg)	71.44 ± 12.12	71.73 ± 12.07	70.78 ± 12.23	0.0784	0.350
Laboratory data					
TC (mmol/L)	4.11 ± 0.97	3.94 ± 1.09	3.87 ± 1.18	0.0626	0.443
TG (mmol/L)	1.39 ± 0.72	1.43 ± 1.02	1.30 ± 0.95	0.1302	0.133
LDL-C (mmol/L)	2.36 ± 0.84	2.21 ± 0.77	2.11 ± 0.76	0.1304	0.109
HDL-C (mmol/L)	1.13 ± 0.36	1.15 ± 0.31	1.15 ± 0.39	0	0.803
eGFR (ml/min/1.73 m^2^)	70.85 ± 23.11	72.16 ± 24.16	67.97 ± 20.35	0.1818	0.031
FPG (mmol/L)	7.05 ± 4.13	6.91 ± 4.45	7.34 ± 3.32	−0.1041	0.224
UA (μmol/L)	351.59 ± 149.66	355.77 ± 164.12	342.34 ± 110.98	0.0897	0.285
Albumin (g/L)	37.54 ± 5.98	39.85 ± 4.22	32.45 ± 6.17	1.5073	<0.001
GNRI	102.47 ± 11.06	107.93 ± 7.31	90.38 ± 7.94	2.3365	<0.001
LVEF (%)	55.65 ± 9.91	56.73 ± 9.23	53.28 ± 10.92	0.3525	<0.001
Comorbidity data					
T2DM	231 (34.89%)	157 (34.43%)	74 (35.92%)	0.0313	0.709
Hypertension	511 (77.19%)	363 (79.61%)	148 (71.84%)	0.1849	0.028
Stroke	138 (20.85%)	94 (20.61%)	44 (21.36%)	0.0184	0.827
Prior MI	120 (18.13%)	82 (17.98%)	38 (18.45%)	0.012	0.886
Hyperlipidemia	151 (22.81%)	111 (24.34%)	40 (19.42%)	0.1175	0.162
CKD	78 (11.78%)	43 (9.43%)	35 (16.99%)	0.2364	0.005
Smoking	164 (24.77%)	112 (24.56%)	52 (25.24%)	0.0158	0.851
Medication data					
Aspirin	642 (96.98%)	444 (97.37%)	198 (96.12%)	0.0731	0.384
Clopidogrel	632 (95.47%)	435 (95.39%)	197 (95.63%)	0.0114	0.892
Statins	615 (92.90%)	426 (93.42%)	189 (91.75%)	0.0651	0.438
β-blocker	418 (63.14%)	286 (62.72%)	132 (64.08%)	0.0282	0.737
ACEI/ARB	364 (54.98%)	252 (55.26%)	112 (54.37%)	0.018	0.830
Coronary lesions					
LAD lesions	560 (84.59%)	385 (84.43%)	175 (84.95%)	0.0145	0.863
LCX lesions	383 (57.85%)	256 (56.14%)	127 (61.65%)	0.1116	0.184
RCA lesions	429 (64.80%)	282 (61.84%)	147 (71.36%)	0.1991	0.018
LM lesions	109 (16.47%)	73 (16.01%)	36 (17.48%)	0.0395	0.638
Multivessel lesions	466 (70.39%)	306 (67.11%)	160 (77.67%)	0.2314	0.006
Gensini score	53.65 ± 42.65	50.89 ± 41.63	59.77 ± 44.31	−0.209	0.013
Treatment				0.0158	0.851
Intensive medication	241 (36.40%)	167 (36.62%)	74 (35.92%)		
PCI	405 (61.18%)	279 (61.18%)	126 (61.17%)		
CABG	16 (2.42%)	10 (2.19%)	6 (2.91%)		

ACEI, angiotensin-converting enzyme inhibitor; ARB, angiotensin receptor blocker; BMI, body mass index; CABG, coronary artery bypass grafting; CKD, chronic kidney disease; DBP, diastolic blood pressure; eGFR, estimated glomerular filtration rate; FBG, fasting blood glucose; GNRI, geriatric nutritional risk index; HDL-C, high-density lipoprotein-cholesterol; LAD, left anterior descending artery; LCX, left circumflex artery; LDL-C, low-density lipoprotein-cholesterol; LM, left main coronary artery; LVEF, left ventricular ejection fraction; MI, myocardial infarction; PCI, percutaneous coronary intervention; RCA, right coronary; SBP, systolic blood pressure; TC, total cholesterol; T2DM, diabetes mellitus type 2; TG, triglycerides; UA, uric acid.

**Figure 2:**
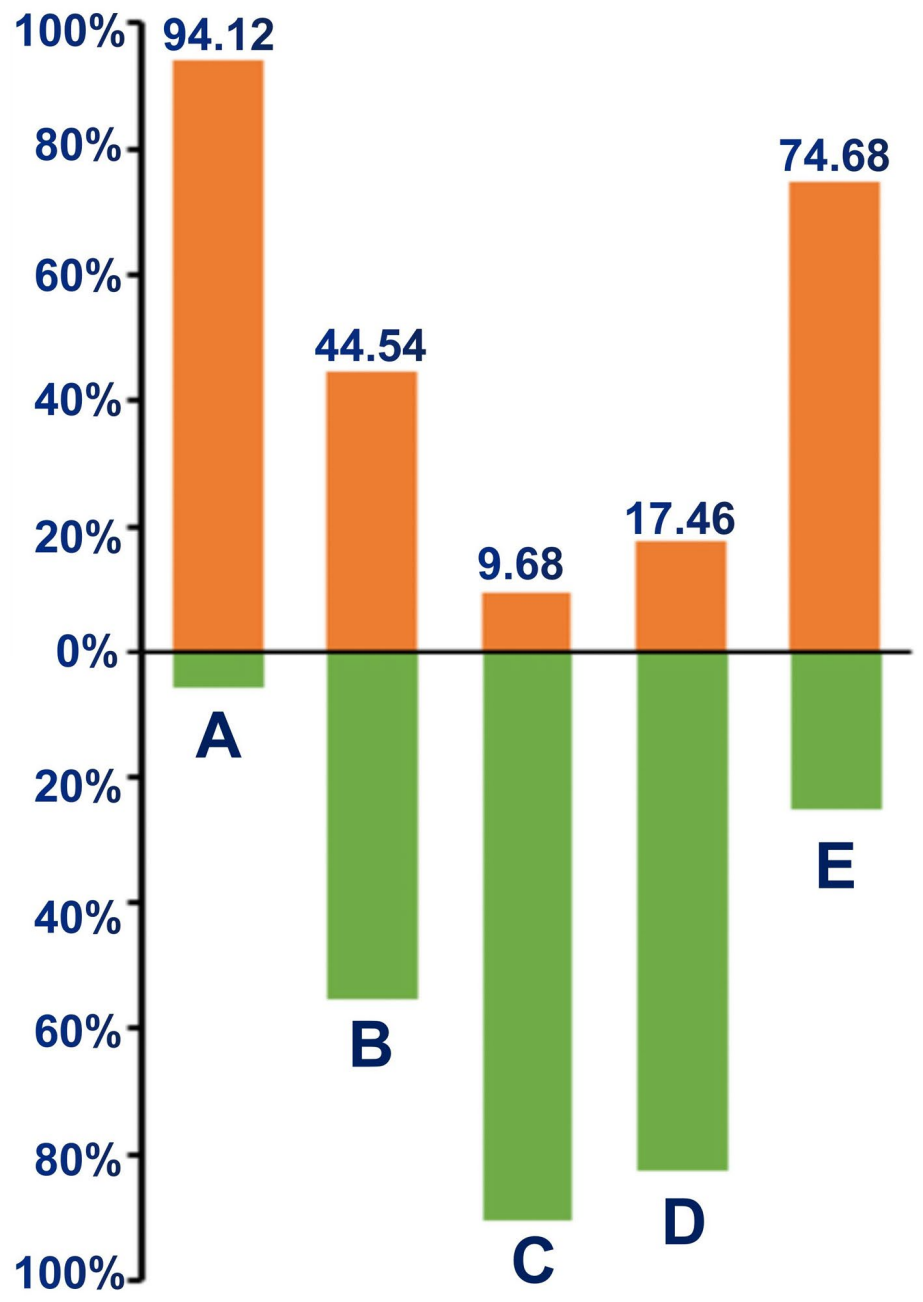
Percentage of malnutrition according to BMI and albumin. **(A)** Under-weight (BMI <18.5 kg/m^2^); **(B)** Normal-weight (BMI 18.5–24.9 kg/m^2^); **(C)** overweight (BMI >24.9 kg/m^2^); **(D)** normal-albumin (≥35 g/L); **(E)** hypo-albumin (<35 g/L).

We further analyzed GNRI in patients with different BMI and albumin levels (Figure 2). The high prevalence of malnutrition was found in patients with BMI <18.5 kg/m^2^ (94.12%), and patients with albumin<35 g/L (74.68%). What is more, there were substantial malnutritional patients in the normal-to over-weight groups.

### 3.2. Association between GNRI and all-cause mortality

The participants were followed for a median of 63 months (IQR 51–74). There were 201 endpoint-events during the follow-up, with 82 occurring in the low GNRI group and 119 in the other. As a nutritional screening index, GNRI was associated with many traditional cardiovascular risk factors (Supplementary Table 1). In univariate analysis (Table 2), a strong positive association was found between all-cause mortality and age, diabetes, stroke, CKD, FPG, Gensini scores, LM lesions, and multivessel lesions. Meanwhile, aspirin, HDL-C, eGFR, LVEF, albumin, and GNRI (HR = 0.97, 95% CI [0.96, 0.99]) were closely associated with a reduction in all-cause mortality. However, male, hypertension, BMI, TC, and LDL-C did not show a significant association with the outcome.

**Table 2:** Univariate Cox regression analyses for independent variables associated with all-cause mortality.

Variable	All-cause mortality	
	HR (95% CI)	*p* value
Male	1.05 (0.77, 1.43)	0.777
Age (year)	1.08 (1.02, 1.15)	0.012
BMI (kg/m^2^)	0.96 (0.92, 1.00)	0.084
SBP (mmHg)	0.99 (0.99, 1.00)	0.058
DBP (mmHg)	0.99 (0.98, 1.01)	0.337
TC (mmol/L)	1.09 (0.98, 1.20)	0.101
TG (mmol/L)	1.06 (0.95, 1.19)	0.314
LDL-C(mmol/L)	1.05 (0.88, 1.26)	0.594
HDL-C(mmol/L)	0.53 (0.33, 0.83)	0.006
eGFR(ml/min/1.73m^2^)	0.98 (0.97, 0.98)	<0.001
FPG (mmol/L)	1.05 (1.03, 1.06)	<0.001
UA (μmol/L)	1.00 (1.00, 1.00)	0.004
albumin	0.96 (0.94, 0.98)	<0.001
GNRI	0.97 (0.96, 0.99)	<0.001
LVEF	0.96(0.95, 0.98)	<0.001
T2DM	1.46 (1.10, 1.93)	0.008
Hypertension	1.07 (0.76, 1.49)	0.712
Stroke	1.50 (1.09, 2.04)	0.012
Prior MI	1.05 (0.75, 1.49)	0.764
Hyperlipidemia	0.81 (0.57, 1.15)	0.230
CKD	2.30 (1.62, 3.25)	<0.001
Smoking	1.08 (0.78, 1.48)	0.648
Aspirin	0.38 (0.21, 0.70)	0.002
Clopidogrel	0.60 (0.34, 1.05)	0.071
Statins	0.98 (0.59, 1.64)	0.951
β-blocker	1.06 (0.80, 1.42)	0.672
ACEI/ARB	1.11 (0.84, 1.48)	0.450
LM lesions	1.53 (1.09, 2.14)	0.014
Multivessel lesions	1.44 (1.04, 2.00)	0.030
Gensini score	1.01 (1.00, 1.01)	<0.001

ACEI, angiotensin-converting enzyme inhibitor; ARB, angiotensin receptor blocker; BMI, body mass index; CKD, chronic kidney disease; DBP, diastolic blood pressure; eGFR, estimated glomerular filtration rate; FBG, fasting blood glucose; GNRI, geriatric nutritional risk index; HDL-C, high-density lipoprotein-cholesterol; LDL-C, low-density lipoprotein-cholesterol; LM, left main coronary artery; LVEF, left ventricular ejection fraction; MI, myocardial infarction; SBP, systolic blood pressure; TC, total cholesterol; T2DM, diabetes mellitus type 2; TG, triglycerides; UA, uric acid.

**Figure 3:**
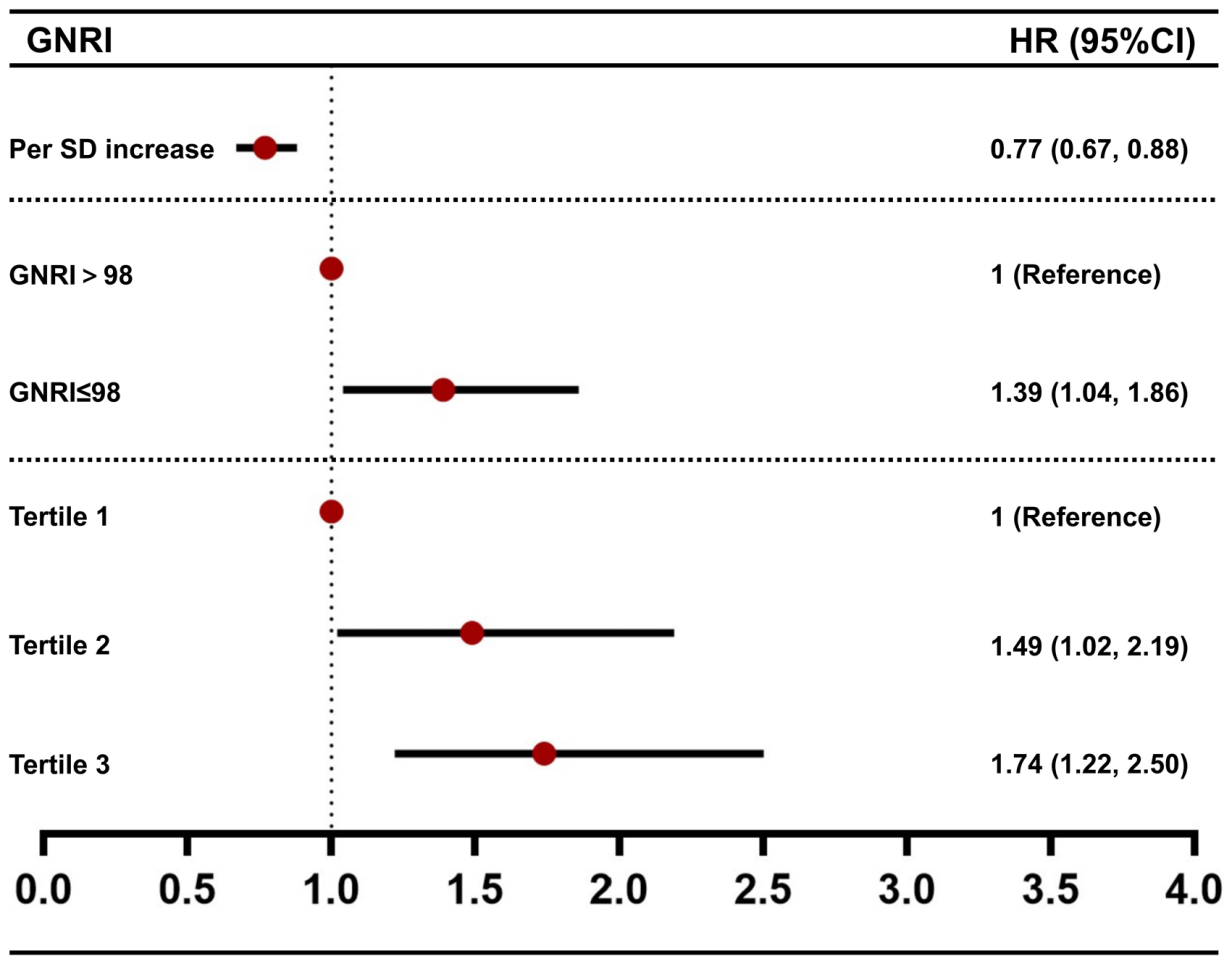
Forest plot of the association between GNRI and all-cause mortality. HR values were from model 3 of Table 3.

We further assessed the association between GNRI and all-cause mortality by multivariable Cox regression analysis (Table 3 and Figure 3). For each SD increase in GNRI, the risk of all-cause mortality was lowered by 23%. And when compared with the high GNRI group, the HR of all-cause mortality in the low GNRI group was 1.39 (95% CI [1.04, 1.86], *p* < 0.05). Then we divided the patients into three groups according to the tertiles of GNRI. Compared with tertile 1, the HRs of tertile 2 and tertile 3 were 1.49 (95% CI [1.02, 2.19], *p* < 0.05) and 1.74 (95% CI [1.22, 2.50], *p* < 0.01), respectively. Subsequently, the trend test revealed a dose–response between GNRI and all-cause mortality.

**Table 3:** Multivariable Cox regression analyses for the association between GNRI and all-cause mortality.

GNRI	Deaths, *N* (%)	HR (95% CI)		
		Model 1	Model 2	Model 3
Per SD increase	201 (28.8%)	0.75 (0.66, 0.85)***	0.76 (0.67, 0.86)***	0.77 (0.67, 0.88)***
>98	119 (26.1%)	1 (Reference)	1 (Reference)	1 (Reference)
≤98	82 (39.8%)	1.70 (1.28, 2.25)***	1.69 (1.27, 2.23)***	1.39 (1.04, 1.86)*
Tertile 1	51 (23.1%)	1 (Reference)	1 (Reference)	1 (Reference)
Tertile 2	62 (28.2%)	1.34 (0.92, 1.94)	1.33 (0.92, 1.93)	1.49 (1.02, 2.19)*
Tertile 3	88 (39.8%)	2.03 (1.43, 2.86)***	1.99 (1.41, 2.82)***	1.74 (1.22, 2.50)**
*p* for trend		<0.001	<0.001	0.003

* *p* < 0.05,

** *p* < 0.01 and

*** *p* < 0.001.

Model 1: unadjusted; Model 2: adjusted for age and sex; Model 3: adjusted for sex, age, diabetes, stroke, CKD, aspirin, eGFR, FPG, UA, LVEF, Gensini score, LM lesions, and multivessel lesions.

### 3.3. Survival analyses of GNRI and all-cause mortality

**Figure 4:**
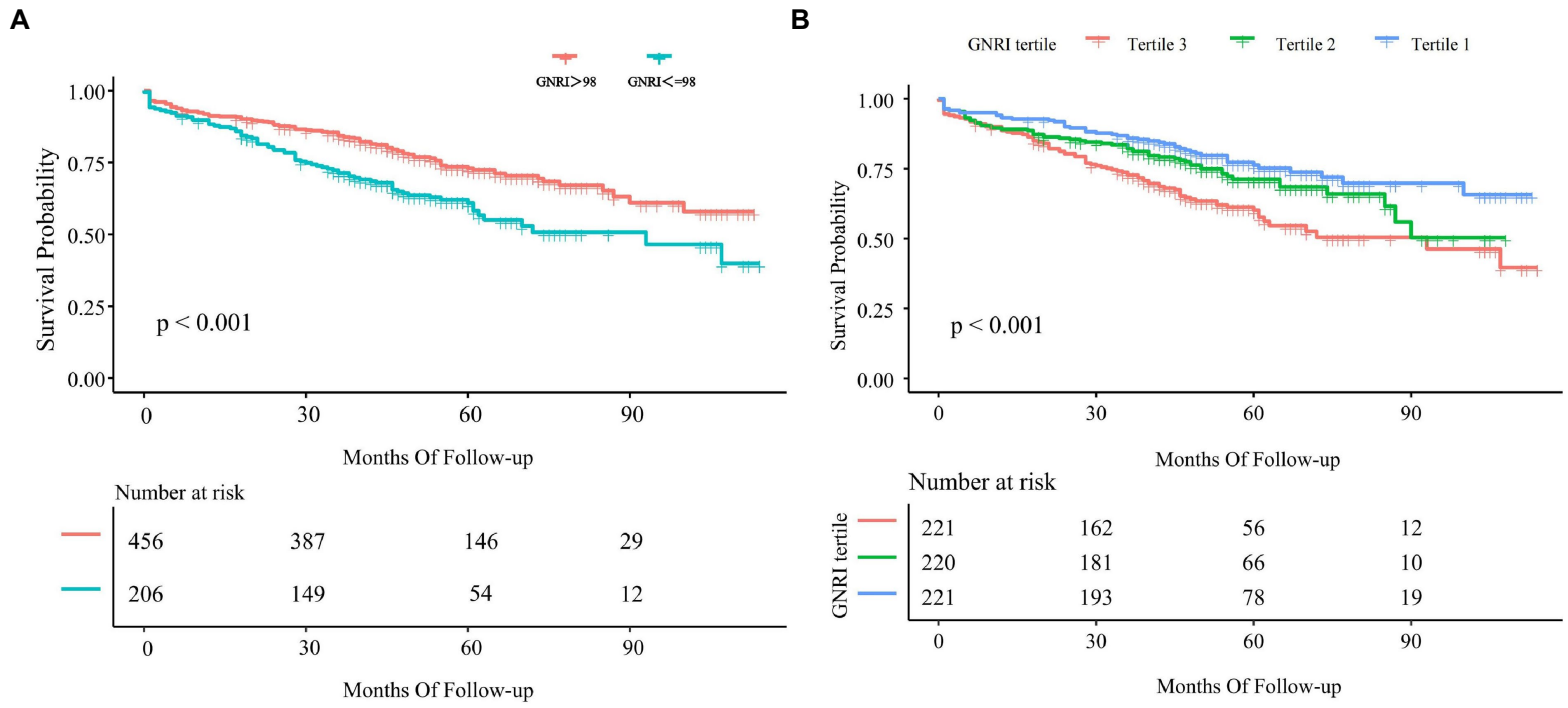
Kaplan–Meier curve of GNRI and all-cause mortality. **(A)** Kaplan–Meier survival curve for all-cause mortality by GNRI 98. **(B)** Kaplan–Meier curve for all-cause mortality by tertiles of GNRI. The result was shown as the Kaplan–Meier curve and *p*-value.

According to the Kaplan–Meier survival analysis (Figure 4A), we found that patients with high GNRI had a longer survival time (*p* < 0.001). Then we further divided GNRI into the tertiles and analyzed them. As shown in Figure 4B, all-cause mortality in tertile 3 was significantly lower than in tertile 1 and tertile 2 (*p* < 0.001).

### 3.4. Subgroup analyses

**Figure 5:**
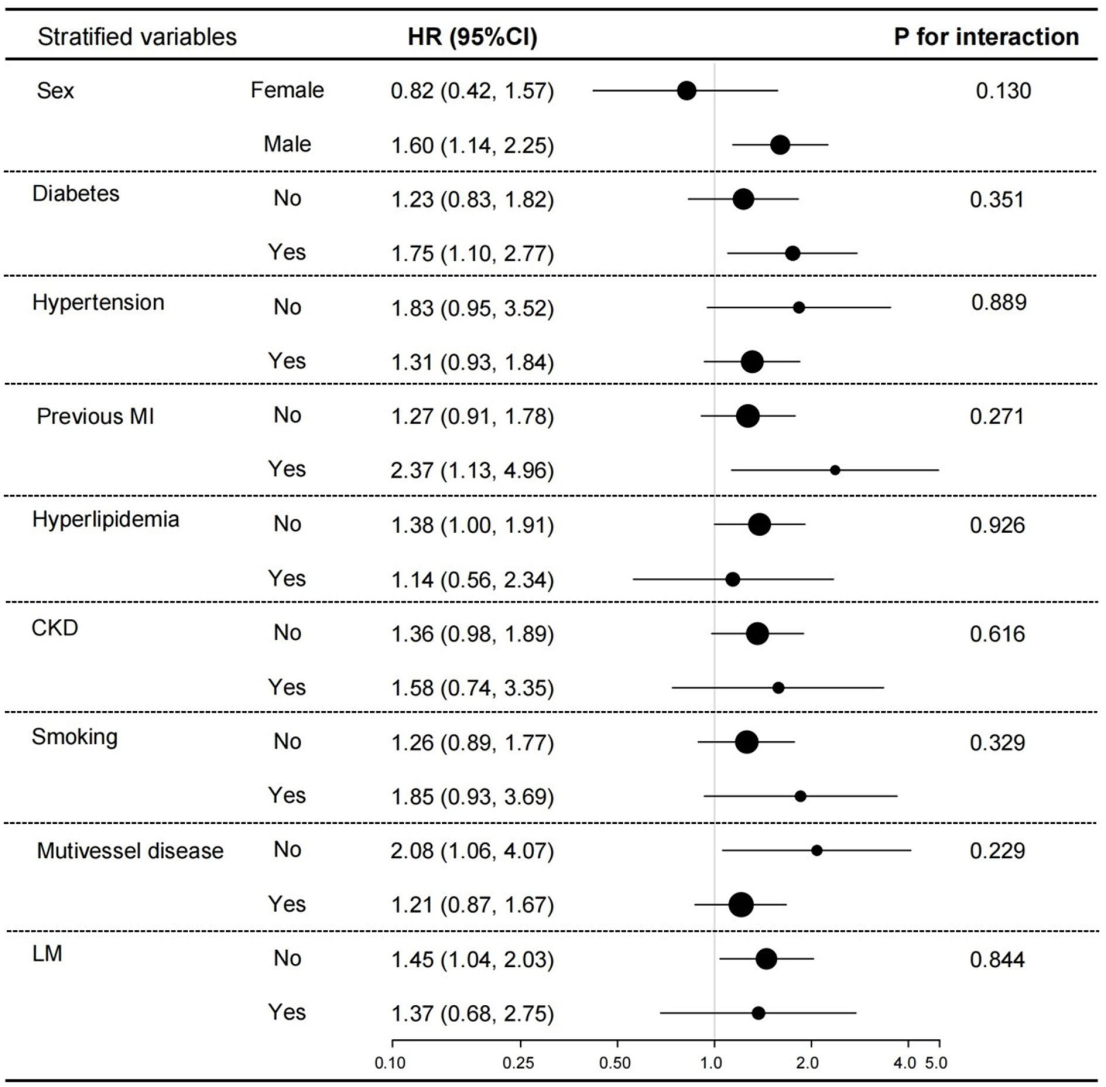
Subgroup analyses for the association between GNRI and all-cause mortality. Adjusted sex, age, diabetes, stroke, CKD, aspirin, eGFR, FPG, UA, LVEF, Gensini score, LM lesions, multivessel lesions, and HDL-C.

In the subgroup analysis (Figure 5), we adjusted sex, age, diabetes, stroke, CKD, aspirin, eGFR, FPG, UA, LVEF, Gensini score, LM lesions, multivessel lesions, and HDL-C except for the stratified variables. The results showed that low GNRI was associated with increased all-cause mortality in those males, with diabetes mellitus, with previous MI, without multivessel lesions, and without LM lesions. But the values of *p* for interaction were all >0.05, suggesting the inverse association between low GNRI with all-cause mortality across all those subgroups.

## 4. Discussion

In this study, we collected clinical data from 662 oldest-old patients with ACS who received coronary angiography and followed for 10 years. As far as we know, this’s the first study to investigate the association between GNRI and all-cause mortality in the oldest-old patients with ACS. Our key findings were the following: (1) the prevalence of malnutrition risk was high in the oldest-old patients with ACS; (2) as a reliable tool for assessing the nutritional status of the elderly, GNRI is associated with many traditional cardiovascular risk factors; (3) low GNRI is an independent risk factor of all-cause mortality in a dose-dependent manner; (4) GNRI has a stable predictive ability for all-cause mortality; and (5) GNRI is a prospective indicator to stratify the risk of all-cause mortality in the oldest-old patients with ACS.

Aging and diseases, especially cardiovascular diseases, are the risk factors for malnutrition (30–32). The oldest-old (elderly >80 years old) tend to have a higher malnutrition risk than those 65–80 years old (33). Despite its high prevalence and negative impact on short-and long-term prognosis, malnutrition remains underdiagnosed. One of the reasons is the lack of a broadly acknowledged definition and diagnostic criterion, and assessing patients’ nutritional status in an emergency like ACS is even more challenging. GNRI seems a promising index because that it can be readily calculated using three objective measurements: serum albumin concentration, height, and body weight. GNRI was strongly associated with poor outcomes in elderly emergency surgery patients, according to Jia et al. (34). Therefore, we picked GNRI as the screening tool for malnutrition in the oldest-old patients with ACS and further assess the association between GNRI and the prognosis of the oldest-old ACS patients.

Serum albumin and BMI are common nutrition indicators; however, they are affected by dehydration, heart failure, inflammation, and other factors (9, 35). In comparison, GNRI is more reliable. It is not simply an overlap of albumin and BMI. Adding GNRI to a baseline model of established risk factors increased the predictive effect of mortality beyond BMI or serum albumin (36, 37). GNRI performed much better than serum albumin level alone in predicting MACE in patients receiving the PCI with rotational atherectomy in the previous study (38). Lots of studies have been conducted to investigate the relationship between GNRI and the prognosis of some chronic diseases, such as chronic heart failure, CKD, and tumors. And the results revealed that low GNRI correlated with longer hospital stays and high mortality. However, few studies evaluated the association between GNRI and the prognosis of elderly patients with ACS, let alone the oldest-old patients. To our knowledge, this was the first time to confirm that a low GNRI was an independent predictor of long-term prognosis in the oldest-old patients with ACS.

Common nutrition screening tools include subjective global assessment (SGA), mini nutritional assessment (MNA), MNA-short form (MNA-SF), prognostic nutritional index (PNI), controlling nutritional status (CONUT), etc. (35, 39). These methods are sensitive to subjective biases and have limitations in terms of time, personnel, and the potential for overdiagnosis. Several studies have compared GNRI with them (40, 41). Wafaa et al. (25) demonstrated that GNRI had a stronger predictive value for describing and classifying nutritional status and nutritional-related problems in elderly hospitalized patients. The GNRI was created as a nutrition-related risk index to recognize and anticipate nutritional-related problems. In the present study, we noticed that a low GNRI at admission was a robust predictor for all-cause mortality in the oldest-old individuals with ACS.

In our study, the GNRI was found to have a positive correlation with BMI, eGFR, LVEF, and serum albumin concentration. The roles of abnormal glucose and lipid metabolism in the pathogenesis of ACS have been widely acknowledged (26). Surprisingly, we did not find an association like in other studies (36, 37, 42) between GNRI and FPG, TC, TG, LDL-C, or HDL-C. Our distinct subjects may explain the differences. As we all know, malnutrition is more common in the elderly due to decreased appetite, impaired digestion and absorption ability, and the impact of diseases (43). So, in our oldest-old patients, hyperglycemia and hyperlipidemia were relatively less prevalent. And our patients followed prescriptions more strictly (92.90% vs. 78.7%) so that maintained their superior blood glucose and cholesterol levels. CKD is a severe risk factor for coronary artery disease (CAD) (44, 45). Patients with CKD generated atherosclerotic plaque as a result of traditional (hyperlipidemia, hypertension, diabetes mellitus, smoking, and so on) and non-traditional (uremia-related cardiovascular disease risk factors such as inflammation, aberrant calcium-phosphorus metabolism, and so on) risk factors (46, 47). As eGFR declines, this progress gets even worse. Herein our study, we discovered the correlation between GNRI and eGFR which may further inspire researchers to explore the relationship between nutritional status and cardio-renal syndrome.

Obesity has been considered a traditional risk factor for cardiovascular diseases. Herein the present study, we found that there were substantial malnutritional patients in the normal-to over-weight patients who may be seen as relatively strong before, and our findings may provide evidence supporting the existence of the obesity paradox (48, 49). Inflammation is a crucial factor in the development of ACS, and it also makes a significant contribution to malnutrition. The malnutrition-inflammation-atherosclerosis (MIA) syndrome has already been proposed as a crucial component of geriatric syndrome (50–52). GNRI was strongly associated with the progression of atherosclerosis in elderly CAD patients (53). In the present study, we found that patients with a low GNRI were more prone to developing multivesicular lesions and got a higher Gensini score. This was the first to report an association between GNRI and the location and severity of the culprit coronary arteries.

There have been reported that effective nutritional interventions could significantly reduce the length of hospital stay and mortality of malnutritional patients (54, 55). By using GNRI, one can quickly recognize the risk of malnutrition and then take measures to improve nutritional status, and the prognosis gets ultimately enhanced. The GNRI was created to assess and forecast nutritionally associated problems. In multi-variate analysis and subgroup analysis, we demonstrated that GNRI stably predicts all-cause mortality of the oldest-old patients in a dose-manner. Based on these, we concluded that the GNRI could serve as a perspective index for risk-stratification and secondary prevention of the oldest-old patients with ACS.

### 4.1. Strengths and limitations

This was the first time enrolled oldest-old patients with ACS, assessed their nutritional status with GNRI on admission, and followed for 10 years. Then we evaluated the association between GNRI and long-term all-cause mortality. In the end, we demonstrated that GNRI is a nutrition assessing tool with prominent advantages, and it may be a hopeful tool to quickly and accurately evaluate nutritional status and predict their prognosis. The results of our study were reliable and practical for clinical use.

However, there were some deficits inevitably. First, this was a single-center cohort study and all participants were Chinese and our was something old, so some selection bias existed. Second, we did not record dietary intake, physical activity, and other factors during the follow-up that may affect nutritional status. Finally, we took 98 as the cut-off value of GNRI, it may need further adjustment according to race or age, etc.

## 5. Conclusion

In this study, we confirmed that low GNRI was an independent predictive factor for all-cause death in the oldest-old patients with ACS, and the relationship between the two was dose-dependent. GNRI may be a perspective index for risk stratification in the oldest-old patients with ACS.

## Data availability statement

The raw data supporting the conclusions of this article will be made available by the authors, without undue reservation.

## Ethics statement

The studies involving human participants were reviewed and approved by Chinese PLA General Hospital’s Ethics Service Center. The patients/participants provided their written informed consent to participate in this study.

## Author contributions

YL, JS, and XH: conducted research and wrote the paper. YL and YS: analyzed the data. YJ, JW, and HL: conducted research. ZF: designed research and had primary responsibility for final content. All authors contributed to the article and approved the submitted version.

## Conflict of interest

The authors declare that the research was conducted in the absence of any commercial or financial relationships that could be construed as a potential conflict of interest.

## Publisher’s note

All claims expressed in this article are solely those of the authors and do not necessarily represent those of their affiliated organizations, or those of the publisher, the editors and the reviewers. Any product that may be evaluated in this article, or claim that may be made by its manufacturer, is not guaranteed or endorsed by the publisher.
