# Supplementary material for: Geriatric nutritional risk index predicts all-cause mortality in the oldest-old patients with acute coronary syndrome: A 10-year cohort study
Source: Front Nutr. 2023 Mar 7;10:1129978. doi: 10.3389/fnut.2023.1129978 (PMC10027908; doi:10.3389/fnut.2023.1129978)
Supplement: Supplementary file 1 [file Table_1.DOCX]

**Supplementary table. Correlation analysis between GNRI and traditional cardiovascular risk factors**

| **Variable** | **Correlation coefficient** | ***P* value** |
| --- | --- | --- |
| **Age（year）** | -0.0576 | 0.139 |
| **BMI (kg/m^2^)** | 0.5928 | <0.001 |
| **SBP (mmHg)** | 0.1110 | 0.004 |
| **DBP (mmHg)** | 0.0492 | 0.206 |
| **Albumin (g/L)^3^** | 0.8129 | <0.001 |
| **TC (mmol/L)** | 0.0202 | 0.605 |
| **TG (mmol/L)** | 0.0759 | 0.051 |
| **LDL-C (mmol/L)** | 0.0663 | 0.088 |
| **HDL-C (mmol/L)** | -0.0404 | 0.300 |
| **UA (umol/L)** | 0.0578 | 0.137 |
| **FPG (mmol/L)** | -0.0286 | 0.463 |
| **eGFR (ml/min/1.73m^2^)** | 0.1111 | 0.004 |
| **LVEF (%)** | 0.1216 | 0.002 |
| **Gensini score** | -0.0540 | 0.165 |
